# Complexation of the Antineoplastic Docetaxel with γ‑Cyclodextrin Significantly Improves Its Solubility and *In Vitro* Safety

**DOI:** 10.1021/acsomega.5c10596

**Published:** 2026-06-02

**Authors:** Thiago S. Sampaio, Natália S. Mendonça, Fabíola V. Carvalho, Fabiano Yokaichiya, Margareth K. K. D. Franco, Luis Fernando Cabeça, Marcos Aurélio Teixeira, Giovana R. Tofoli, Maria Florencia Martini, Eneida de Paula

**Affiliations:** 1 Department of Biochemistry and Tissue Biology, Institute of Biology, 124594 University of Campinas (UNICAMP), Campinas, SP 13083-862, Brazil; 2 Department of Physics, Federal University of Paraná (UFPR), Curitiba, PR 81531-980, Brazil; 3 119500 Nuclear and Energy Research Institute, IPEN, CNEN/SP, São Paulo, SP 05508-000, Brazil; 4 Department of Chemistry, Tecnológica Federal University of Paraná (UTFPR), Londrina, PR 86036-370, Brazil; 5 Faculdade São Leopoldo Mandic, Instituto de Pesquisa São Leopoldo Mandic, Campinas, SP 13045-755, Brazil; 6 Instituto Tecnológico de Buenos Aires (ITBA), National Scientific and Technical Research Council (CONICET), Buenos Aires C1437, Argentina

## Abstract

Docetaxel (DTX) is a frontline taxane for breast cancer, but its poor aqueous solubility, limited intestinal permeability, and rapid clearance compromise therapeutic efficacy. Moreover, the commercial formulation Taxotere induces severe side effects, including hypersensitivity reactions, neutropenia, neuropathy, musculoskeletal toxicity, and nasolacrimal duct stenosis. Here, we investigated γ-cyclodextrin (γ-CD) complexation as a strategy to enhance DTX performance. A DTX:γ-CD inclusion complex was obtained by cosolubilization and lyophilization, reaching equilibrium within 5 h at 25 °C, with an optimal 1:2 stoichiometry, but such determinations were hindered by variability in the measurements, indicating that the supramolecular association of DTX and γ-CD was not straightforward. Complex formation was supported by thermal, spectroscopic, and microscopic analyses, and it provided a 20-fold increase in docetaxel solubility. Molecular dynamics simulations confirmed the 1:2 DTX:γ-CD stoichiometry, revealing sequential binding of two γ-CD to the DTX carbamate group along with additional noninclusion interactions. Cytotoxicity assays in human umbilical vein endothelial cells (HUVEC) and HS578T breast cancer cells showed that the DTX:γ-CD complex preserved the antitumor activity of docetaxel across all tested concentrations while reducing toxicity in endothelial cells. At the highest concentration (0.1 mM), HUVEC viability remained close to 50% with the DTX:γ-CD complex, compared with only ∼20% for the commercial formulation, indicating a substantial protective effect without compromising cytotoxic efficacy against HS578T tumor cells. Overall, γ-CD complexation substantially improves DTX solubility and safety, supporting its potential as a promising alternative delivery system to enhance docetaxel’s therapeutic index.

## Graphical abstract

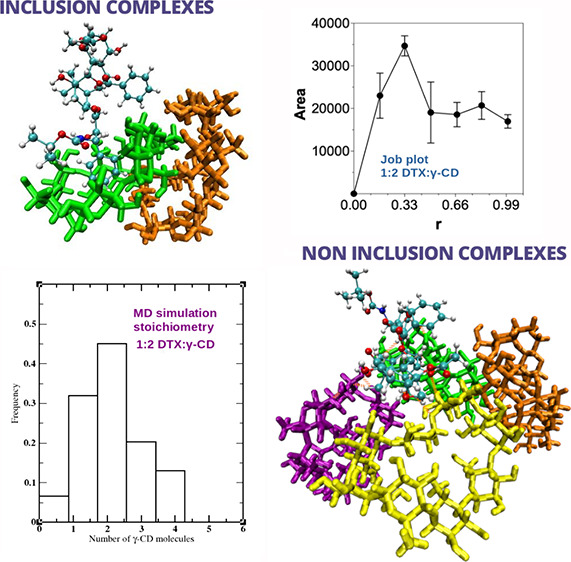

## 1 Introduction

Docetaxel (DTX) is a semisynthetic derivative of 10-deacetyl-baccatin III, a taxane compound extracted from the leaves of the yew tree (*Taxus baccata* L).^1^ It was introduced in 1996 for the treatment of anthracycline-refractory metastatic breast cancer, and its indications were subsequently extended to include neoplasms of the skin, lung, prostate, stomach, head and neck, among others.^2^ Despite its broad antineoplastic potential, the clinical application of DTX remains constrained by its classification as a Biopharmaceutics Classification System (BCS) class IV drug,^3^ characterized by poor aqueous solubility (∼5 mg/L or 6 μM), low intestinal permeability and rapid systemic clearance.^4^ Indeed, the major challenges with taxane drugs such as DTX lie in their low bioavailability and inherent toxicity.^5^ To achieve solubilization, they require surfactants (e.g., Polysorbate-80 in Taxotere), which introduce additional adverse effects—including hypersensitivity reactions, neutropenia, neuropathy, musculoskeletal toxicity, and nasolacrimal duct stenosis—thereby compounding their overall toxicity.^5,6^

To mitigate these drawbacks and enhance both the bioavailability and anticancer efficacy of DTX, we explored its inclusion complexation with cyclodextrins (CDs). In the pharmaceutical field, CDs are widely employed as complexing agents to increase the aqueous solubility of poorly soluble drugs, thereby improving their bioavailability and physicochemical stability. CDs possess a hydrophilic outer surface and a hydrophobic central cavity, allowing them to encapsulate appropriately sized and polar molecules within their macrocyclic structure to form host–guest inclusion complexes.^7^ This complexation can improve the drug’s solubility, enhance its stability, and extend its release profile. The stability of such complexes is primarily mediated by noncovalent interactions, including hydrophobic forces, H-bonds, steric effects, and van der Waals interactions.^8^ CDs are also attractive due to their commercial availability, biocompatibility, low immunogenicity, and established safety profiles—features particularly relevant to formulations for cancer therapy.

Several types of CDs are available for pharmaceutical applications. Among them, γ-cyclodextrin (γ-CD) is approved by the FDA for administration via infiltrative routes.^9^ Among natural CDs, γ-CD possesses a considerably larger internal cavity than β-cyclodextrin, the most widely used CD, making it particularly suitable for encapsulating bulky molecules such as DTX. The encapsulation of antineoplastic agents in γ-CD has shown promise—for instance by enhancing doxorubicin solubility.^10^ Although DTX has been previously complexed with modified β-CD derivatives, such as hydroxypropyl-sulfobutyl β-CD^11^ and cationic and PEGylated β-CD,^12^ to the best of our knowledge, no study has yet reported the complexation of DTX with γ-CD.

This study aimed to develop a novel, surfactant-free, formulation for the anticancer agent docetaxel through complexation with γ-CD. A comprehensive characterization of the DTX:γ-CD complex was conducted using experimental approaches—including Job plot analysis, UV–vis spectroscopy, differential scanning calorimetry (DSC), X-ray diffraction (XRD), Infrared (FTIR), Scanning electron microscopy (SEM), Nuclear Magnetic Resonance (^1^H NMR)—and *in silico* modeling—including structural minimization, parametrization and molecular dynamics simulations studies—followed by evaluation of the formulation’s *in vitro* cytotoxicity.

## 2 Materials and Methods

### 2.1 Materials

Docetaxel powder (DTX) was a gift from Cristália Prod. Quím. Farmac. (Itapira, Brazil); DTX in solution—a generic form from Taxotere—was donated by Blau Farmacêutica (Cotia, Brazil); γ-cyclodextrin was donated from Cyclolab (Hungary); ultrapure water was obtained with the Milli-Q system (Merck KGaA, Darmstadt, Germany); absolute ethanol was purchased from Dinâmica Quim. (Brazil); methanol for HPLC was purchased from Merck Millipore (Germany); RPMI-1640 medium, Dulbecco’s Modified Eagle Medium (DMEM), fetal bovine serum (FBS), 3-(4,5-dimethylthiazol-2-yl)-2,5-diphenyltetrazolium bromide (MTT), penicillin, streptomycin sulfate and trypsin were supplied by Sigma Chem. Co. (St. Louis, MO, USA); human breast cancer cells (HS578T, ATCC HTB-126) and umbilical vein endothelial cells (HUVEC, ATCC PCS-100-010) were purchased from American type culture collection (ATCC, Manassas, VA, USA); Dimethyl sulfoxide (DMSO) was purchased from Laborclin (Pinhais, Brazil).

### 2.2 Methods

#### 2.2.1 DTX:γ-CD Inclusion Complexation

DTX:γ-CD complex was prepared with 12.3 mM (half the clinical dose, respectively 20 mg/mL or 24.75 mM) of docetaxel and 24.6 mM γ-CD. Docetaxel was diluted in ethanol (10 mL) and cyclodextrin was diluted in deionized water (10 mL). After solubilization of the compounds, the solutions were mixed and maintained under a magnetic stirrer for equilibration. Then, the sample was dried in a rotary evaporator (Heidolph Hei-VAP Value Digital rotary evaporator), resuspended in water and lyophilized (Lyofast S12: IMA lyophilizer). DTX was quantified either by its absorption in the UV–vis region, at 232 nm^13^ or by high-performance liquid chromatography (HPLC), see Section 2.2.2. The study was carried out at 25 °C and the equilibration time was defined by the time in which the absorbance stabilizes, for more than 2 h.^14^

#### 2.2.2 DTX Quantification and Assessment of the HPLC Analytical Method

DTX was quantified by high-performance liquid chromatography (HPLC) with UV detection under the conditions described in Table 1. The analytical method was adapted from Gao et al.^15^ Analyses were performed using a Waters Breeze 2 High-Performance Liquid Chromatograph (Waters, Milford, MA, USA).

**Table 1:** Chromatographic Conditions for Docetaxel Quantification

parameter	condition
column	C18 5 μM x 150 × 4.6 mm (Gemini)
oven temperature	30 °C
mobile phase	water:methanol 30:70 (v/v)
flux	1 mL·min^–1^
injection volume	10 μL
wavelength	230 nm

To validate the analytical method, the following parameters were evaluated: limit of quantification, limit of detection, and linearity. Linearity was assessed to demonstrate that the results obtained with the analytical method were directly proportional to the DTX concentration in the sample within a specified range, adopting a correlation coefficient (*r* ^2^) of 0.990 as the minimum acceptable criterion, and requiring that the slope be significantly different from zero.^16^ Seven different concentrations (2.5–50 μg/mL DTX), analyzed in triplicate on three consecutive days, were used to construct the calibration curve. Linear regression was performed using the least-squares method.^16,17^

The limit of detection (LD) and the limit of quantification (LQ) were determined using eqs 1 and 2, respectively, where SD is the standard deviation (*y*-intercept) and IC is the slope of the mean analytical curve.

$$\mathrm{LD}=\frac{\mathrm{SD}\times 3}{\mathrm{IC}}$$ 1

$$\mathrm{LQ}=\frac{\mathrm{SD}\times 10}{\mathrm{IC}}$$ 2

#### 2.2.3 Determination of Complex Stoichiometry

For the Job Plot experiment, stock solutions of DTX and γ-CD were prepared. From them, 10 dilutions were made to a final volume of 10 mL in deionized water, to a constant final concentration of 6 μM ([DTX] + [γ-CD]). After stirring for 24 h, the content of each tube was read at 230 nm and the changes in areas were plot as a function of *r* (the mole fraction of the guest molecule), according to eq 3:^18,19^

$$r=\frac{\left[\mathrm{DTX}\right]}{\left[\mathrm{DTX}]+[\mathrm{\gamma ‐}\mathrm{CD}\right]}$$ 3

where [DTX] and [γ-CD] refers to docetaxel and γ-cyclodextrin concentrations.

#### 2.2.4 Solubility Experiments

In this work, solubility was evaluated by adding DTX or the DTX:γ-CD complex to deionized water until a supersaturated solution was obtained, characterized by the presence of a precipitate at the bottom of the vial. The experiments were then conducted using ten different concentrations of a DTX solution and the DTX:γ-CD complex, with each solution being analyzed in triplicate.^20^ The samples were kept on a shaker for 5 h, under agitation (200 rpm) and centrifuged for 15 min. Afterward, each sample was promptly filtered (through a 0.45 μm pore membrane) and DTX was quantified by HPLC, as described in Section 2.2.2.^21,22^ Calibration curves were constructed using the data obtained and the solubility limit of the compound was determined at the inflection point of the curves.

#### 2.2.5 Differential Scanning Calorimetry

Differential scanning calorimetry (DSC) was employed to assess cyclodextrin inclusion complexes through comparison of the thermal behavior of the individual components, their physical mixture, and the resulting inclusion complex. Samples of DTX, γ-CD, the DTX:γ-CD physical mixture, and the inclusion complex were analyzed using a DSC 2910 instrument (TA Instruments, New Castle, USA) at the Instituto de Química, UNICAMP. Samples were sealed in aluminum pans and heated from 25 to 250 °C at a rate of 10 °C/min under a dynamic nitrogen atmosphere (50 mL min^–1^). The instrument was calibrated using an indium standard, and thermal data were processed with Thermal Solutions software (v.1.25).^23^

#### 2.2.6 X-ray Diffraction

X-ray powder diffraction (XRD) data were obtained in a XRD7000 diffractometer (Shimadzu, Tokyo, Japan), using a Cu–Kα source (λ­(kα) = 1.5406 Å) at a scanning step of 2° min^–1^, between values of 2θ (5–90°), detector was set to 40 kV and the irradiation source to 20 mA. Samples of DTX, γ-CD, DTX:γ-CD physical mixture and complex were analyzed.

#### 2.2.7 Fourier Transform Infrared

The Fourier transform InfraRed (FTIR) spectroscopy of the sample of DTX, γ-CD, DTX:γ-CD physical mixture, and DTX:γ-CD complex was performed at the Instituto de Química (UNICAMP) using the Agilent Carry 630 (Australia) attenuated total reflectance Fourier transform infrared spectrophotometer (ATR-FTIR), USA. The specifications of the measurements were: 26 transmittance mode, 128 scans per analysis and resolution of up to 2.0 cm^–1^, in the range of 4000 to 400 cm^–1^.^24^

#### 2.2.8 Scanning Electron Microscopy

Scanning electron microscopy (SEM) was employed to analyze the morphology of the samples. Freeze-dried samples of DTX, γ-CD, their physical mixture, and the DTX:γ-CD inclusion complex were mounted on aluminum stubs using double-sided carbon tape. The mounted specimens were sputter-coated with gold under vacuum for 200 s using a Bal-Tec SCD-050 sputter coater to render them electrically conductive. Imaging was performed using a JSM-5800LV microscope (JEOL, Japan) operating in secondary electron mode at an accelerating voltage of 15 kV.^25^

#### 2.2.9 ^1^H Nuclear Magnetic Resonance

Hydrogen Nuclear magnetic resonance (^1^H NMR) experiments were performed at CNPEM using an Agilent DD2 spectrometer operating at a proton resonance frequency of 499.726 MHz. The experiments were conducted at 25 °C, using TMSP-*d*_4_ (3-(trimethylsilyl)-2,2′,3,3′-tetradeuteropropionic acid) as the internal reference (0 ppm). The residual deuterium signal was used for field locking and shimming to optimize magnetic field homogeneity. DTX was solubilized in 4% deuterated dimethyl sulfoxide (DMSO-*d*_6_). Diffusion-ordered spectroscopy (DOSY) experiments were performed using 16 different gradient amplitudes, with the diffusion time optimized to 0.06 s. Data were processed using the DOSY Toolbox software. The diffusion coefficient of the complex was determined assuming exchange between the free and complexed states of the ligand. The complexed molar fraction (*f*_complex_) and the association constant (*K*_a_) were calculated from the diffusion coefficients (*D*) of the guest (γ-CD), host (DTX) and the complex (DTX:γ-CD) using eqs 4 and 5, respectively.^14^

$$f_{\mathrm{compl}\mathrm{ex}}=\frac{\left(D_{\mathrm{free}}-D_{\mathrm{complex}}\right)}{\left(D_{\mathrm{free}}-D_{\mathrm{host}}\right)}$$ 4

$$K_{a}=\frac{f_{\mathrm{complex}}}{\left((1-f_{\mathrm{complex}})([\mathrm{host}]-f_{\mathrm{complex}}[\mathrm{guest}])\right)}$$ 5

#### 2.2.10 Molecular Dynamics Simulations

Atomic-scale molecular dynamics (MD) simulations of DTX and γ-CD in aqueous solution were performed using periodic boundary conditions (pbc) to account for collective intermolecular interactions. The simulated system consisted of 8 DTX molecules, 8 γ-CD molecules, and 32,000 water molecules contained in a cubic simulation box with a volume of 998 nm^3^. Molecular structures were built using ChemAxon Marvin software, which was also used to analyze physicochemical and theoretical descriptors.

System assembly and MD simulations were carried out using the GROMACS 2020.6 software package.^26,27^ The initial configuration was generated with a random molecular distribution using the *gmx insert-molecules* tool. The GROMOS 96 54a7 force field^28,29^ was applied to both DTX and γ-CD, while water molecules were modeled using the simple point charge (SPC) model. All components were assigned partial charges corresponding to pH 7.

Electrostatic interactions were treated using the smooth particle mesh Ewald (SPME) method,^30,31^ with a real-space cutoff of 1.5 nm, a grid spacing of 0.24 nm, and cubic interpolation. van der Waals interactions were truncated at 1.5 nm. Simulations were performed in the NPT ensemble using the V-rescale thermostat, with the system coupled to a heat bath at 298 K and a relaxation time of 0.1 ps. Pressure was maintained at 1 atm using an isotropic C-rescale barostat. A time step of 1 fs was used for integration of the equations of motion.

Prior to the production run, the system underwent four equilibration stages of 2 ns each, with progressive temperature increases. The ground-state geometry of DTX was optimized using density functional theory^32^ with the B3LYP functional and the 6-31G+(d,p) basis set. Partial atomic charges were derived from single-point HF/6-31G+(d,p) calculations using Gaussian 16,^33^ following the Merz–Singh–Kollman scheme.^34^ Force constants and intermolecular parameters were assigned by analogy with structurally similar compounds described within the GROMOS force field.

γ-CD parameters were generated using the Automated Topology Builder version 2.2 (https://atb.uq.edu.au), followed by manual correction. Production MD simulations were carried out for 230 ns after equilibration, analyzing two independent replicas. Trajectory visualization and snapshot generation were performed using VMD (https://www.ks.uiuc.edu/Research/vmd/) and Grace Softwares (https://plasma-gate.weizmann.ac.il/Grace/).

#### 2.2.11 Cell Viability Assays

Human umbilical vein endothelial cells (HUVEC, ATCC PCS-100–010) and human breast cancer cells HS578T (ATCC HTB-126) were used in this study. No primary cell cultures were employed. HUVECs were cultured in RPMI medium supplemented with 10% fetal bovine serum (FBS) and 1% penicillin–streptomycin, whereas HS578T cells were cultured in DMEM containing 10% FBS and 1% penicillin–streptomycin. Both cell lines were maintained at 37 °C in a humidified incubator with 5% CO_2_ until reaching semiconfluency.

Cells were plated in 96-well plates at 2 × 10^4^ cells/well and allowed to adhere for 24 h. Subsequently, the medium was replaced with fresh medium containing treatments (DTX, DTX:γ-CD, or γ-CD) at increasing concentrations: 1.5 × 10^–6^ to 1.2 × 10^–4^ M for docetaxel and 2.4 × 10^–6^ to 2.4 × 10^–4^ M for γ-CD. After 48 h of treatment, the medium was removed, wells were washed with 5 mM PBS, and cell viability was evaluated using the MTT assay.^35^ Briefly, 0.5 mg/mL MTT was added and incubated for 3 h at 37 °C in the dark. Formazan crystals were then solubilized with DMSO, and absorbance was read at 570 nm using an ELx800-GEN5RC plate reader (Life Res. Co., London). Cell viability was expressed as a percentage relative to untreated control cells.

## 3 Results and Discussion

### 3.1 DTX:γ-CD Complex Formation

#### 3.1.1 Time and Stoichiometry of Complexation

The DTX:γ-CD inclusion complex was prepared as described in Methods section. Monitoring DTX absorbance at 230 nm, equilibrium was reached after approximately 5 h (Figure 1A). The stoichiometry of the complex, determined using the Job plot method,^18^ indicated a 1:2 molar ratio of DTX:γ-CD (Figure 1B).

**Figure 1:**
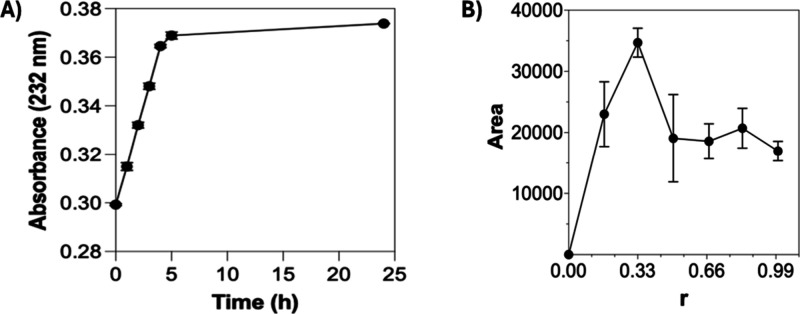
(A) Time required to reach complexation equilibrium. (B) Job plot showing a maximum at *r* = 0.33, indicating a 1:2 DTX:γ-CD molar ratio. Results expressed as mean ± SD (*n* = 3).

The minimum time to reach equilibrium in host–guest inclusion complex formation is a critical parameter that reflects the underlying interaction forces. Based on the data in Figure 1B, and consistent with eq 3,^19^ the maximum r value of 0.33 confirmed the 1 DTX molecule to 2 γ-CD molecules (1:2 DTX:γ-CD) stoichiometry.

#### 3.1.2 Increase in DTX Solubility upon Complexation

The aqueous solubility of DTX increased from 6 μM^4,36^ to 120 μM in the presence of γ-CD (Figure 2). DTX concentrations were quantified by HPLC as detailed in the Methods section.

**Figure 2:**
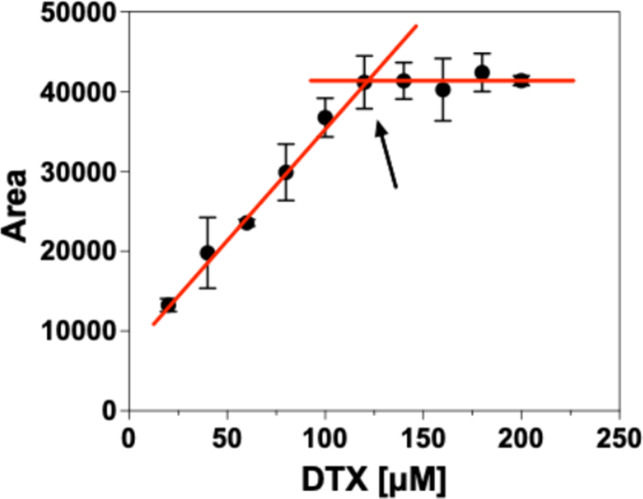
Aqueous solubility of DTX in the complex, at 25 °C. Results expressed as mean ± SD (*n* = 3).

The 20-fold increase in solubility observed upon complexation with γ-CD supports the formation of a molecular inclusion complex, consistent with findings for other host–guest systems.^36^

### 3.2 Solid-State Characterization of the DTX:γ-CD Complex

Characterization of the solid 1:2 DTX:γ-CD complex was performed by DSC, XRD, FTIR and SEM to investigate the interaction between DTX and γ-CD and complex formation.

#### 3.2.1 DTX: γ-CD Characterization by Differential Scanning Calorimetry

Figure 3 presents the DSC thermograms of pure γ-CD, DTX, their physical mixture, and the inclusion complex. γ-CD exhibited an endothermic peak at 203.94 °C, while DTX displayed peaks at 131.6 °C (melting point) and 186.5 °C (degradation) according to the literature.^37^ The physical mixture retained peaks corresponding to the individual components (166.23 and 200.22 °C). In contrast, the thermogram of the 1:2 DTX:γ-CD complex showed the disappearance of DTX’s characteristic peaks, replaced by a single low-intensity endothermic event at 135 °C. The DSC results suggest that DTX amorphization is attributable to the formation of inclusion complexes. Further characterization (by XRD and ^1^H NMR) was performed to confirm this hypothesis.

**Figure 3:**
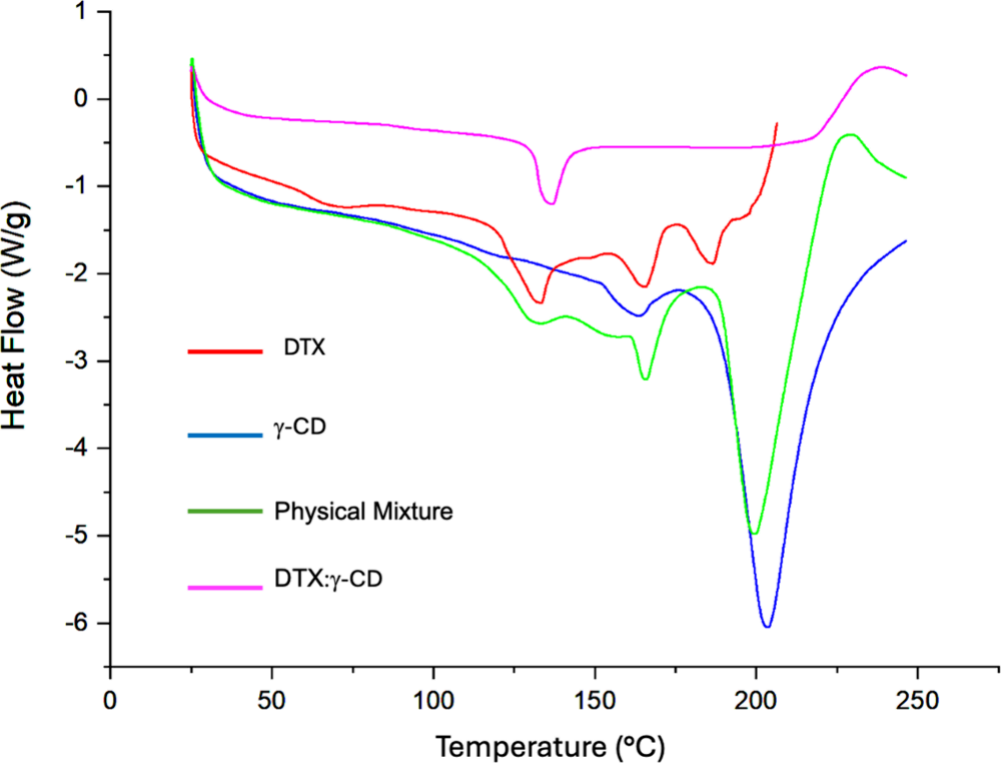
DSC thermograms of DTX, γ-CD, physical mixture (1:2 mol %), and DTX:γ-CD inclusion complex (1:2 mol %).

#### 3.2.2 X-ray Powder Diffraction

Figure 4 and Figure S6 (raw data) show the X-ray diffractograms of the pure compounds, physical mixture, and the DTX:γ-CD complex.

**Figure 4:**
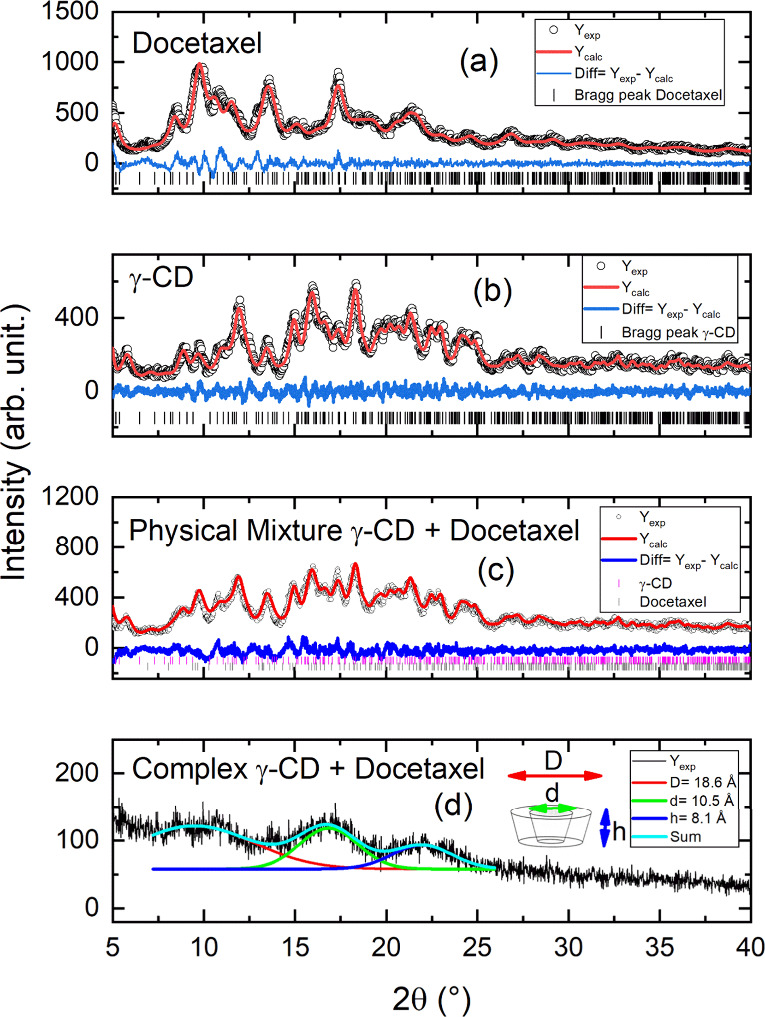
XRD patterns of (a) DTX, (b) γ-CD, (c) physical mixture (1:2 mol %), and (d) DTX:γ-CD inclusion complex (1:2 mol %).

The XRD patterns confirmed the crystalline nature of both DTX (Figure 4a) and γ-CD (Figure 4b). The physical mixture (Figure 4c) exhibited a simple superposition of the individual diffraction profiles. In contrast, the diffractogram of the DTX:γ-CD complex (Figure 4d) no longer displayed the characteristic crystalline peaks of either component, indicating the formation of an inclusion complex. Furthermore, the modeled dimensions of the γ-CD ring within the complex (outer diameter *D* = 18.6 Å, inner diameter *d* = 10.5 Å, and height *h* = 8.1 Å) were larger than those of uncomplexed γ-CD (*D* = 16.9 Å, *d* = 9.5 Å, *h* = 7.8 Å),^38^ supporting the encapsulation of DTX within the γ-CD cavity.

#### 3.2.3 FTIR Spectroscopy of the DTX:γ-CD Complex

Figure 5 displays the FTIR spectra of DTX, γ-CD, their physical mixture, and the inclusion complex. The results obtained reveal significant changes in the absorption bands, especially in the regions corresponding to the C=O stretching, the benzene ring and the C–O bending vibrations, evidencing once again the formation of the inclusion complex.

**Figure 5:**
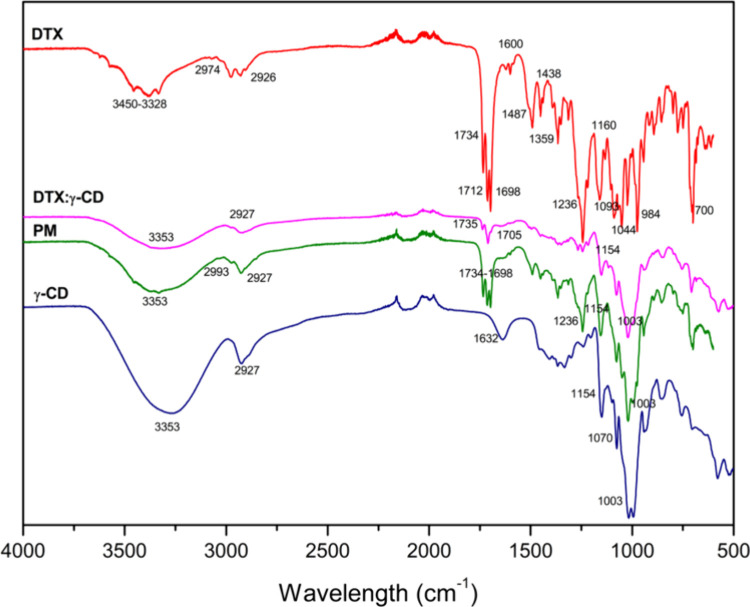
FTIR spectra of DTX, γ-CD, their physical mixture, and the DTX:γ-CD complex (1:2 mol %).

The characteristic C=O stretching vibration band of DTX (1712–1734 cm^–1^) shifted to 1705–1708 cm^–1^ upon complexation. So, this change indicates a strong interaction between the carbonyl group of DTX and γ-CD. In addition, the benzene ring band at 1487 cm^–1^ disappears, indicating the aromatic ring’s involvement in γ-CD cavity inclusion. Other evidence came from the physical mixture spectrum, that resembles a simple overlay of each individual component: DTX, as for the C=O stretching band that appears in the region of 1698–1734 cm^–1^ or the benzene ring band, located at 1487 cm^–1^, or γ-CD, as for the C–O and C–C stretching vibrations of γ-CD at 1003 cm^–1^.

#### 3.2.4 DTX:γ-CD Characterization by SEM

The morphology of the DTX, γ-CD, physical mixture and DTX: γ-CD complex samples was evaluated by SEM (Figure 6). To exclude the influence of freeze-drying in the molecules or complex morphology all samples were submitted to lyophilization prior to SEM analysis. The micrograph of DTX (Figure 6A) reveals a crystalline arrangement, typical of the drug and in agreement with that described in the literature.^39^ In the γ-CD sample (Figure 6B), crystalline particles were also observed, according to the XRD data.

**Figure 6:**
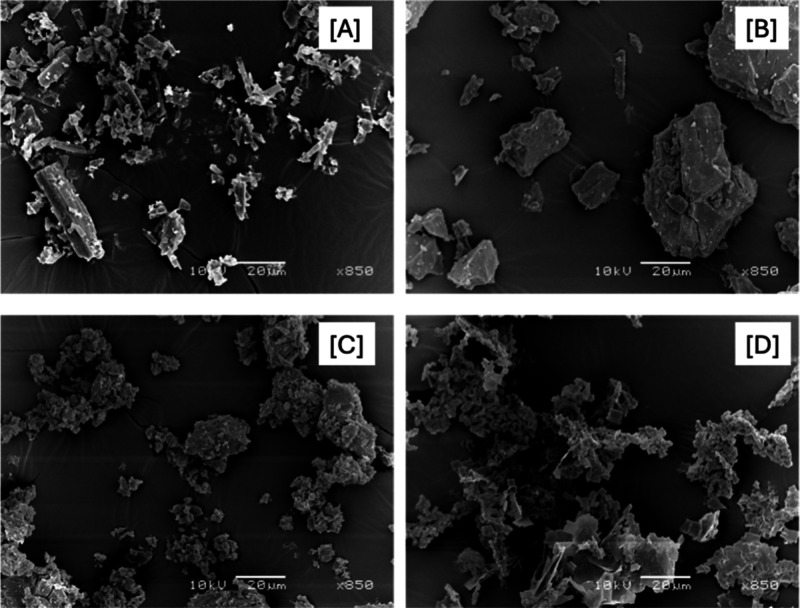
SEM images of (A) pure DTX, (B) pure γ-CD, (C) physical mixture (1:2 mol %), and (D) DTX:γ-CD inclusion complex (1:2 mol %).

In the micrograph of the physical mixture (Figure 6C), crystalline particles (from γ-CD) can be observed mixed with a more irregular distribution of smaller particles, from DTX crystals. Unlike in the micrograph of the DTX:γ-CD inclusion complex (Figure 6D), a sharp change in the appearance of the particles and the absence of the DTX crystal structures reflect the occurrence of a molecular interaction, through the formation of the inclusion complex.

#### 3.2.5 ^1^H NMR Analysis

Through ^1^H NMR analysis of the DTX sample, γ-CD and the DTX: γ-CD complex, it was not possible to observe changes in the chemical shifts of the drug or CD hydrogens. The low aqueous solubility of DTX, along with spectral overlap, may have hindered these measurements. Neither the ^1^H NMR results obtained using the DOSY (Diffusion Ordered Spectroscopy) sequence were more satisfactory. The diffusion coefficients determined pseudo-2D spectrum obtained from DOSY experiments (Figure S1) were 0.9 × 10^–10^ m^2^/s for γ-CD, 1.17 × 10^^–^10^ m^2^/s for DTX and 1.15 × 10^–10^ m^2^/s for the DTX:γ-CD complex. Using eqs 4 and 5, the binding fraction was calculated to be 7%, corresponding to an average association constant of 472 M^–1^.^14,40^

#### 3.2.6 Molecular Dynamics Simulations

The interaction energy (*E*_int_) between the different components of the mixture was calculated based on the contributions of Coulombic and van der Waals forces acting between them. Figure 7 displays the *E*_int_ between the components of the DTX and γ-CD mixture in aqueous solution, as a function of the simulated time. A decrease in the interaction of each component with water can be observed over time, as the interaction between cyclodextrins and DTX molecules increases.

**Figure 7:**
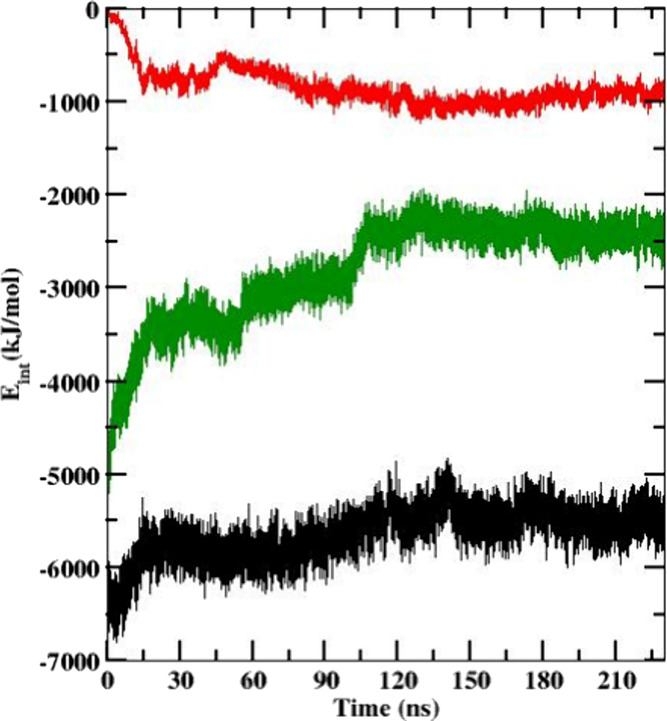
*E*_int_ between DTX-water (black), γ-CD-water (green), and DTX:γ-CD (red).

To determine the average stoichiometry of the formed complex, the number of cyclodextrin molecules located within a 1.5 nm radius from the center of mass of each of the 8 DTX molecules was evaluated over the simulated time (excluding the first 20 ns of the simulation, during which the complexes were stabilizing). Based on these data, a histogram was generated, as shown in Figure 8.

**Figure 8:**
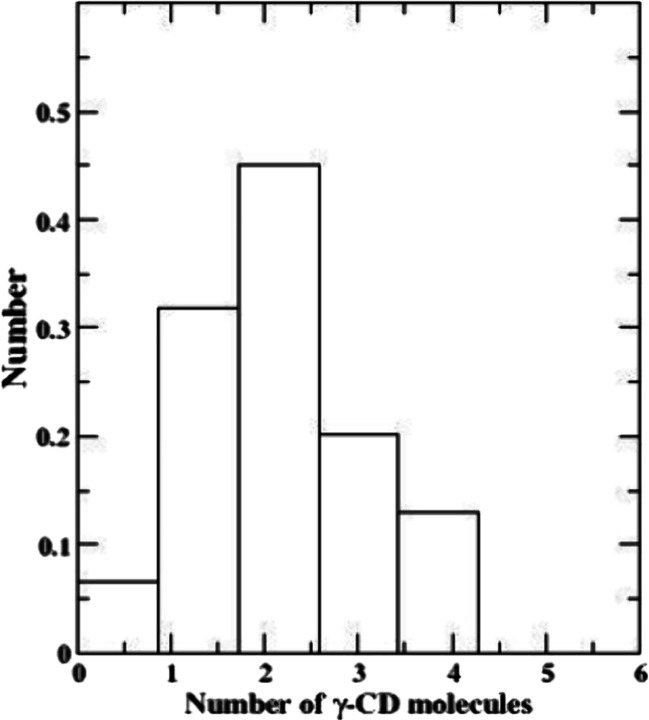
Histogram of the number of CD molecules located within a 1.5 nm radius from the center of mass of each DTX molecule (*n* = 8), excluding the first 20 ns of simulation.

MD (Figure 8) yielded an average stoichiometry in excellent agreement with the experimental Job plot (Figure 1B). However, the Job plot showed absorbance increasing to *r* = 0.33 (DTX:γ-CD ratio 1:2) before decreasing to a half-height plateau—an asymmetry that may reflect noninclusion complexes,^41^ which were also identified by MD (see below).

Analysis of the minimum distances between each DTX molecule and each of the 8 γ-CD molecules revealed that, in 7 out of the 8 DTX molecules, stable structures were formed with one or more γ-CD molecules, which persist for 40 ns or more. In some cases, these complexes remained stable throughout the entire simulation once formed (Figure S2A–H).

Minimum distances (below 4 Å) between these host–guest interactions, when sustained for more than 20 ns, are indicative of complex formation. These complexes may correspond either to inclusion complexes, where the compound enters the cyclodextrin cavity, or to noninclusion complexes, in which stabilization occurs through interactions with the hydroxyl groups on the external surface of the cyclodextrin.^40,41^ In the latter case, although the interaction is stable, the standard deviation of the minimum distance tends to be slightly higher than in inclusion complexes.

A molecular analysis of the simulation trajectories revealed the formation of both types of complexes: inclusion and noninclusion. Inclusion complexes were observed in two out of the eight simulated DTX molecules (Figure S2A,H), with both showing the same positioning of the DTX molecule within the γ-CD cavity. In both cases, the aromatic ring adjacent to the DTX carbamate group entered through the wider rim of the cyclodextrin, and stabilization was favored by the formation of hydrogen bonds between the hydroxyl groups of the CD and the heteroatoms of the carbamate moiety of the DTX (Figure 9A,B).

**Figure 9:**
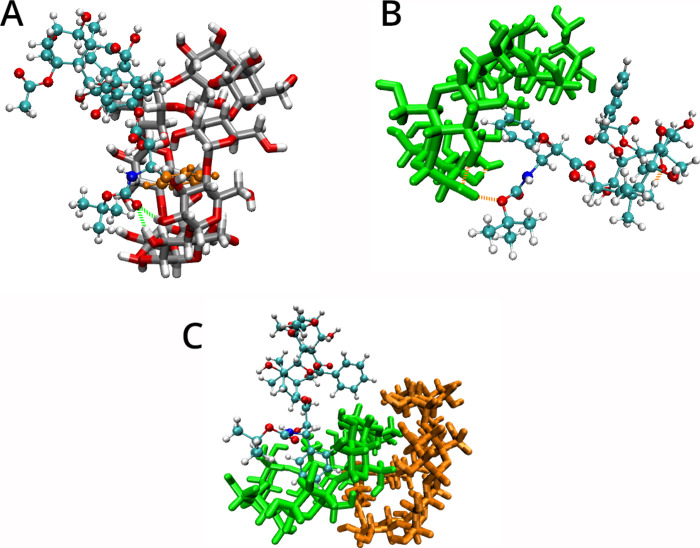
(A, B) Initial, 1:1 DTX:γ-CD inclusion complex, corresponding to Figure S2A and Figure S2H, respectively; (C) final, DTX:γ-CD inclusion complex stabilized by a second cyclodextrin molecule (corresponding to the DTX molecule shown in Figure S2A). In panel (A), the γ-CD is displayed in licorice representation and colored by atom type: C (gray), O (red), and H (white). The DTX molecule is shown in CPK representation, with the same atom-type color scheme except for carbon atoms, which are colored cyan. The aromatic ring adjacent to the carbamate group is highlighted in orange to emphasize its insertion into the CD cavity. Two hydrogen bonds between CD hydroxyl groups and the carbonyl group of the DTX carbamate are shown in light blue. In panel (B), the inclusion complex corresponds to the DTX molecule shown in Figure S2H. The γ-CD is shown in licorice representation and colored green; the DTX molecule is shown in CPK representation and colored by atom type; the hydrogen bond between a CD hydroxyl group and a carbamate oxygen of DTX is highlighted in orange.

Although the inclusion complexes formed in both cases initially exhibited a 1:1 stoichiometry, shortly after formation one or more additional CD molecules became stabilized alongside the complexating cyclodextrin (as shown in Figure 9C). As for the noninclusion complexes formed during the simulation, they exhibited variable stoichiometry, ranging from DTX:γ-CD 1:2 to 1:4. Nonetheless, all of them remained stable over time (see Figure S2B,C,E–G).

To assess the stability of the different inclusion and noninclusion complexes formed between DTX and γ-CD, we computed the *E*_int_ for each DTX molecule (Figure S3) with its nearest cyclodextrin partner, analyzing them separately according to the type of complex: inclusion (Figure S3A) or noninclusion (Figure S3B). The inclusion complexes exhibited substantially stronger interaction energies, ranging from −26 to −30 kcal/mol. By contrast, the noninclusion complexes showed weaker and more heterogeneous interactions, with average values around −15 kcal/mol and larger fluctuations over time. In Figure S3B, the green curve depicts a DTX molecule transitioning from a noninclusion state (thin line) to an inclusion complex (thick line), with its *E*_int_ shifting accordingly to the characteristic energy range of each binding mode.

#### 3.2.7 Cytotoxicity against Breast Cancer and Normal Cells

Figure 10 shows the effects of treating human umbilical vein endothelial cells (HUVEC) and human breast cancer cells (HS578T) for 48 h with commercial DTX or the DTX:γ-CD complex. γ-CD alone did not affect the viability of either HUVEC or HS578T cells, except at the highest concentration tested (2.4 × 10^–4^ M, corresponding to 1.2 × 10^–4^ M in the DTX:γ-CD complex) for HUVECs, indicating that γ-CD is largely biocompatible across most of the evaluated range (Figure S4).

**Figure 10:**
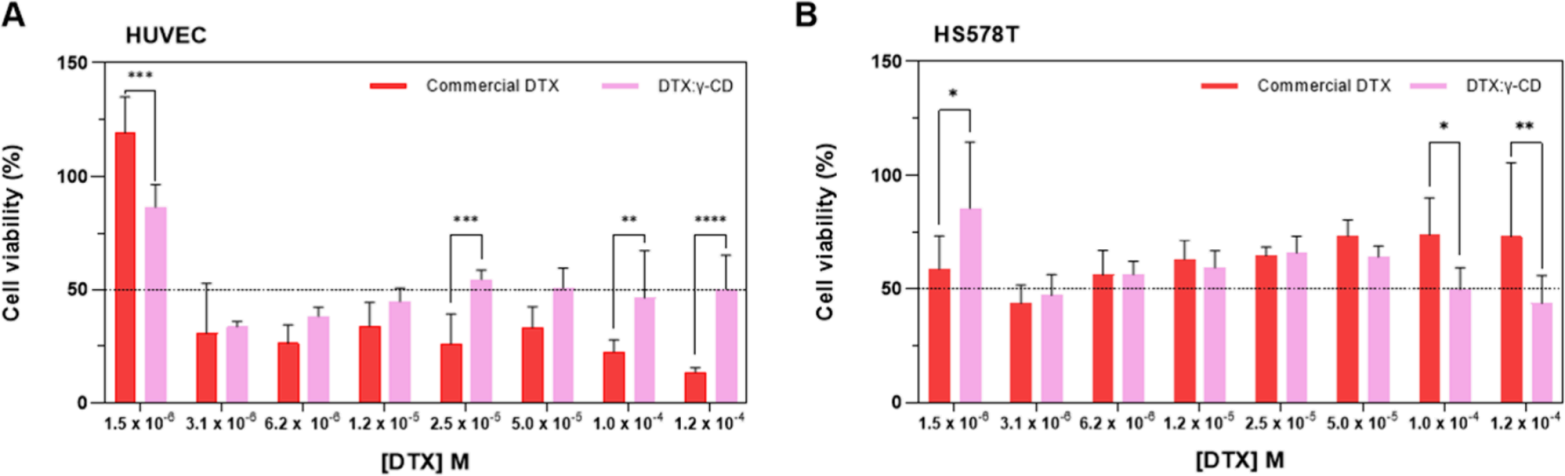
Cell viability (MTT assay) of HUVEC (A) and HS578T cells (B) treated for 48 h with commercial DTX or the DTX:γ-CD complex. Results expressed as mean ± SD (*n* = 6). Statistical analysis by two-way ANOVA plus Tukey–Kramer post hoc: *p < 0.05; **p < 0.01; ***p < 0.001, ****p < 0.0001.

Commercial DTX caused a marked and dose-dependent reduction in endothelial cells’ viability beginning at the lower concentrations (≥3.1 × 10^–6^ M). Importantly, the DTX:γ-CD complex consistently induced significantly less cytotoxicity than commercial DTX, with viability differences ranging from 28% to 37% at intermediate and high concentrations (*p* < 0.001 to *p* < 0.0001). At the highest concentration tested (1.2 × 10^–4^ M), viability in DTX-treated HUVEC cells decreased to approximately 13.5%, whereas cells exposed to the DTX:γ-CD complex maintained viability near 50%, revealing a substantial protective effect conferred by cyclodextrin complexation.

In HS578T breast cancer cells both commercial and DTX:γ-CD complex affected cell viability, with DTX:γ-CD complex being more cytotoxic than the commercial formulation at the highest concentrations tested (25–30% at 1.0–1.2 × 10^–4^ M, *p* < 0.05 and *p* < 0.01, respectively). Only at the lower concentration (1.5 × 10^–6^) the antitumor potency of the complexed DTX was significantly lower than that of commercial DTX (*p* < 0.05).

Together, these findings demonstrate that (i) the cytotoxicity of the DTX:γ-CD complex arises from drug–carrier complexation rather than from the free γ-CD, and (ii) that complexation of docetaxel with γ-CD allows a clear dissociation between endothelial toxicity and antitumor efficacy. The DTX:γ-CD system markedly reduces DTX-induced cytotoxicity in normal endothelial HUVEC while maintaining or enhancing cytotoxic activity against HS578T tumor cells. This dual behavior highlights DTX:γ-CD as a promising delivery strategy to mitigate vascular toxicity without compromising antitumor effectiveness.

## 4 Conclusions

In this study, we combined experimental approaches with molecular simulations to elucidate the interactions underlying DTX:γ-CD complexation. The experimental data indicated a 1:2 guest–host stoichiometry. However, establishing this stoichiometry—as well as determining the equilibrium time and association constant—was challenging, suggesting that the supramolecular association between DTX and γ-CD is complex. To further investigate this behavior, molecular dynamics studies were performed. The simulations confirmed the 1:2 DTX:γ-CD stoichiometry and showed that the benzene and carbamate groups of DTX insert into the wider rim of two γ-CD molecules. Moreover, MD simulations provided evidence of additional noninclusion complexation, stabilized by intermolecular H bonds between the carbamate group and γ-CD. This dual nature of docetaxel−γ-cyclodextrin association and the higher energy associated with inclusion complex formation likely accounts for the even format of the Job plot (Figure 1B).

Overall, complexation with γ-cyclodextrin enabled the development of a surfactant-free pharmaceutical formulation of DTX that significantly enhanced its aqueous solubility—by approximately 20-fold—while reducing cytotoxicity toward normal cells and preserving antitumor efficacy. These findings highlight the potential of the DTX:γ-CD as a strategy to improve the therapeutic index of taxane-based chemotherapy.

## Supplementary Material



## Notes

^1^H NMR: DOSY spectrum of the DTX:γ-CD complex (Figure S1). Molecular dynamics: minimum distances between DTX and γ-CD molecules (Figure S2) and interaction energies for the DTX:γ-CD inclusion complexes (Figure S3). Cytotoxicity assays: effect of γ-CD on the viability of HUVEC and HS578T cells after 48 h of exposure (Figure S4). DSC: individual thermograms of DTX, γ-CD, the DTX:γ-CD physical mixture, and the inclusion complex (Figure S5), as well as the melting point and enthalpy values (Table S1). XRD: raw diffractograms of DTX, γ-CD, the DTX:γ-CD physical mixture, and the inclusion complex (Figure S6) (PDF)
