# Supplementary material for: Complexation of the Antineoplastic Docetaxel with γ‑Cyclodextrin Significantly Improves Its Solubility and In Vitro Safety
Source: ACS Omega. 2026 Jun 2;11(23):33358–67. doi: 10.1021/acsomega.5c10596 (PMC13280900; doi:10.1021/acsomega.5c10596)
Supplement: Supplementary file 1 [file ao5c10596_si_001.pdf]

Complexation of the antineoplastic docetaxel with gamma-cyclodextrin significantly improves its solubility and in vitro safety

Thiago S. Sampaio[1]; Natália S. Mendonça[1]; Fabíola V. Carvalho[1]; Fabiano Yokaichiya[2]; Margareth K.K.D. Franco[3]; Luis Fernando Cabeça[4], Marcos Aurélio Teixeira[5]; Giovana R. Tofoli[5]; Maria Florencia Martini[6]*, Eneida de Paula[1]*

[1]Department of Biochemistry and Tissue Biology, Institute of Biology, University of Campinas (UNICAMP), 13083-862 Campinas/SP, Brazil.
[2]Department of Physics, Federal University of Paraná (UFPR), 81531-980 Curitiba/PR, Brazil.
[3]Nuclear and Energy Research Institute, IPEN, CNEN/SP, 05508-000 São Paulo/SP, Brazil.
[4]Department of Chemistry, Tech. Federal University of Paraná (UTFPR), 86036-370 Londrina/PR, Brazil.
[5]Faculdade São Leopoldo Mandic, Instituto de Pesquisa São Leopoldo Mandic, 13045-755 Campinas/SP, Brazil.
[6]Instituto Tecnológico de Buenos Aires (ITBA), National Scientific and Technical Research Council (CONICET), C1437 Buenos Aires, Argentina.

* Co-corresponding authors:
Eneida de Paula. Department of Biochemistry and Tissue Biology, Institute of Biology, UNICAMP. Rua Monteiro Lobato 255, bl. F sup sala 9, 13083-862, Campinas-SP, Brazil. E-mail: depaula@unicamp.br
M. Florencia Martini. Department of Research and Doctoral Studies, Instituto Tecnológico de Buenos Aires (ITBA), National Scientific and Technical Research Council (CONICET). Iguazú 341, C1437 Buenos Aires, Argentina. E-mail: mmartini@itba.edu.ar

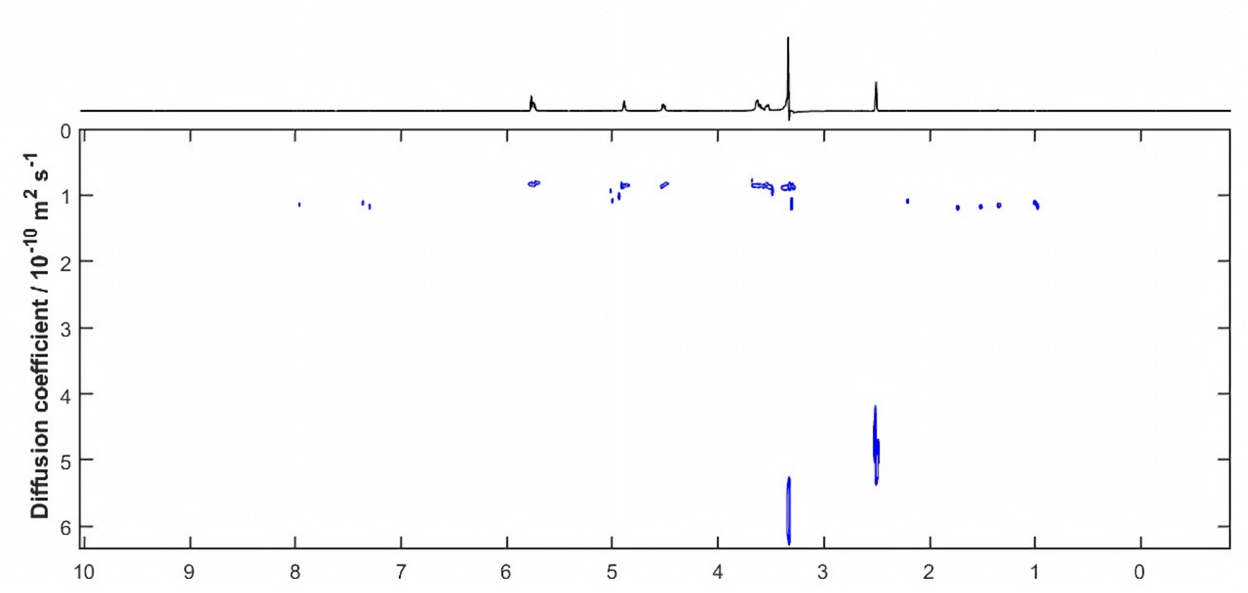

Figure S1. ^{1}H-NMR: DOSY spectrum of the DTX-γ-CD complex.

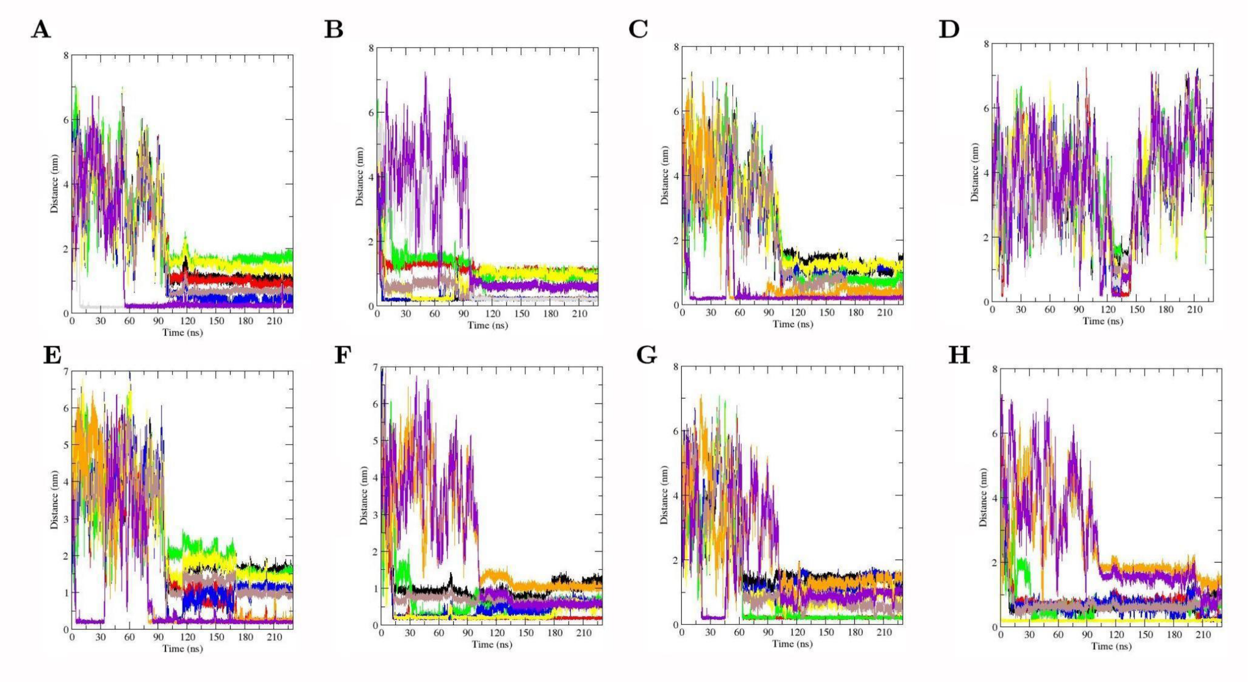

Figure S2. Minimum distance between each of the 8 DTX molecules (A–H) and the γ-CD molecules. In each plot, the γ-CD molecules are represented by eight distinct colors.

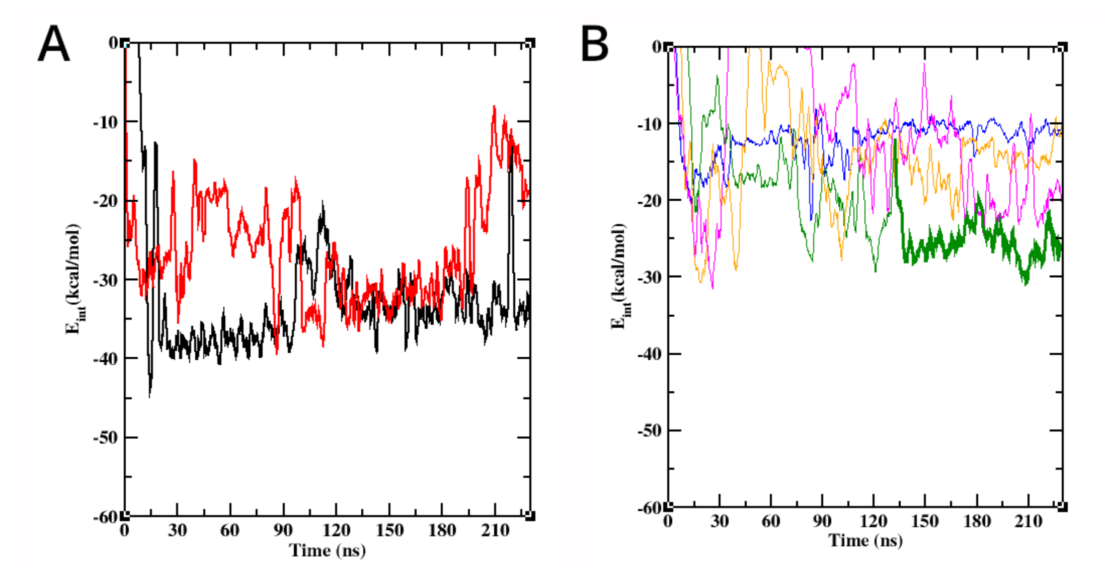

Figure S3. A. Interaction energies for DTX:γ-CD inclusion complexes. **B.** Interaction energies for DTX:γ-CD non-inclusion complexes. The green trace illustrates a DTX molecule transitioning from a non-inclusion state (thin line) to an inclusion state (thicker line), with a corresponding shift in E_{int} into the characteristic range of inclusion complexes.

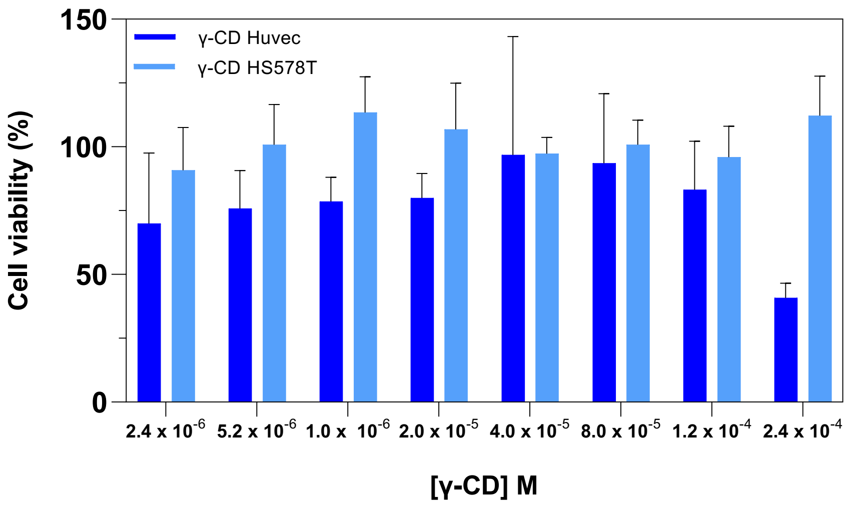

Figure S4. Viability of HUVEC and HS578T cells after 48 h of exposure to γ-cyclodextrin (γ-CD) at different concentrations. Results expressed as mean ± SD (n = 6).

A)

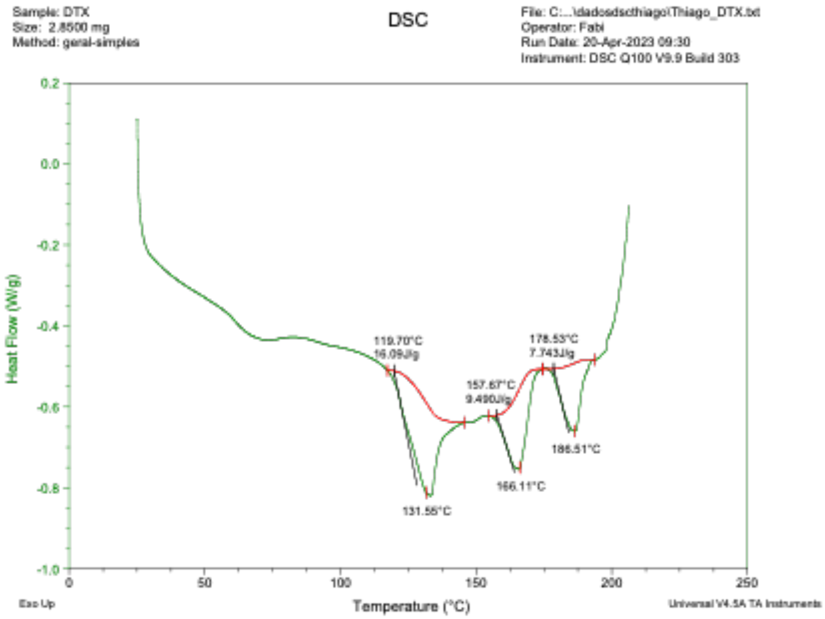
Sample: DTX
Size: 2.8500 mg
Method: geral-simples
DSC
File: C:...\dadosdscthiago\Thiago_DTX.txt
Operator: Fabi
Run Date: 20-Apr-2023 09:30
Instrument: DSC Q100 V9.9 Build 303
Heat Flow (W/g)
119.70°C
16.09J/g
157.67°C
9.490J/g
178.53°C
7.743J/g
131.55°C
166.11°C
186.51°C
Exo Up
Temperature (°C)
Universal V4.5A TA Instruments

B)

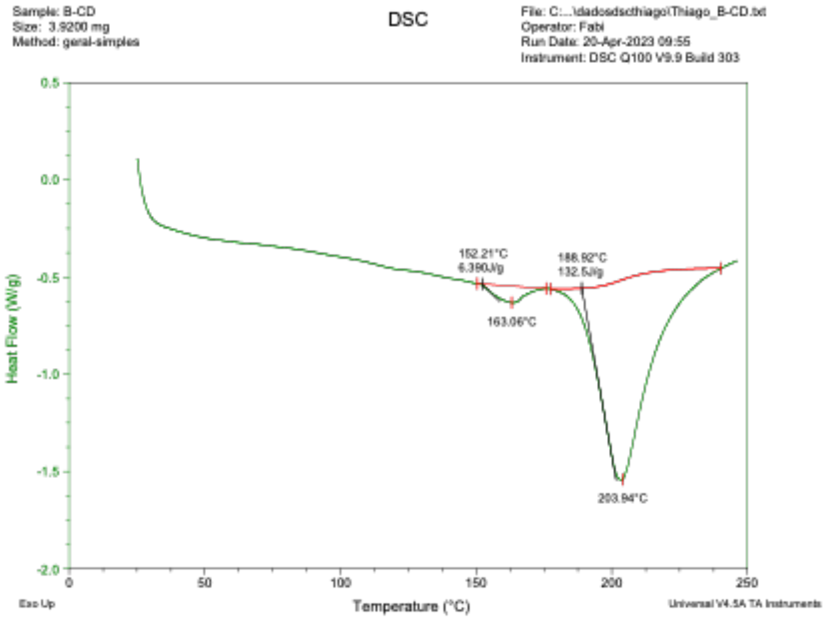
Sample: B-CD
Size: 3.9200 mg
Method: geral-simples
DSC
File: C:...\dadosdscthiago\Thiago_B-CD.txt
Operator: Fabi
Run Date: 20-Apr-2023 09:55
Instrument: DSC Q100 V9.9 Build 303
Heat Flow (W/g)
152.21°C
6.390J/g
188.92°C
132.5J/g
163.06°C
203.94°C
Exo Up
Temperature (°C)
Universal V4.5A TA Instruments

C)

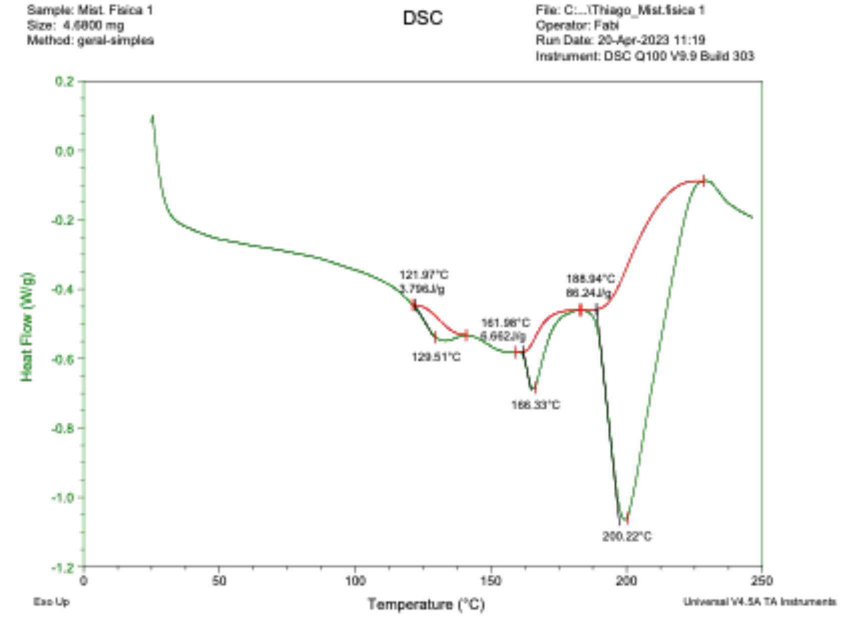

D)

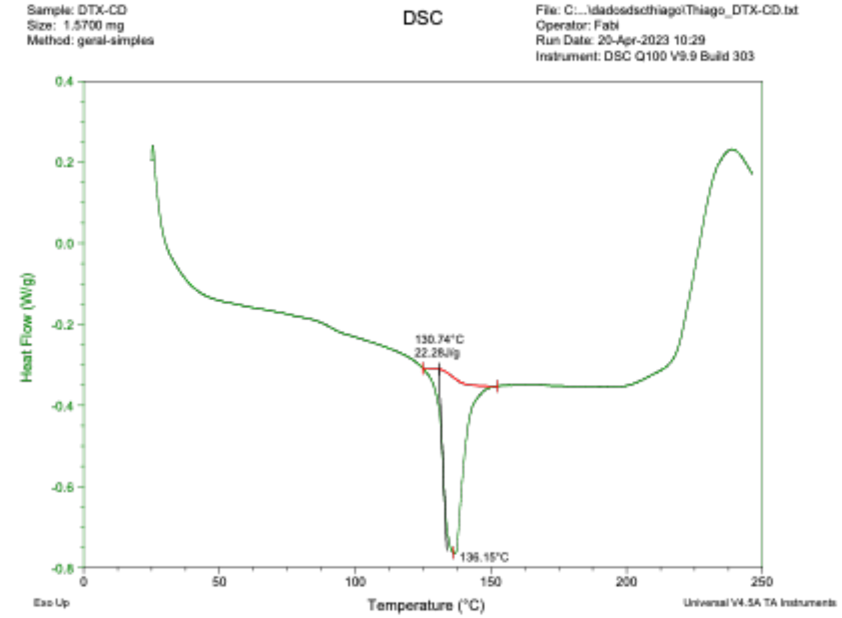

Figure S5. DSC thermograms of (A) DTX, (B) γ-CD, (C) physical mixture (1:2 mol%), and (D) DTX:γ-CD inclusion complex (1:2 mol%).

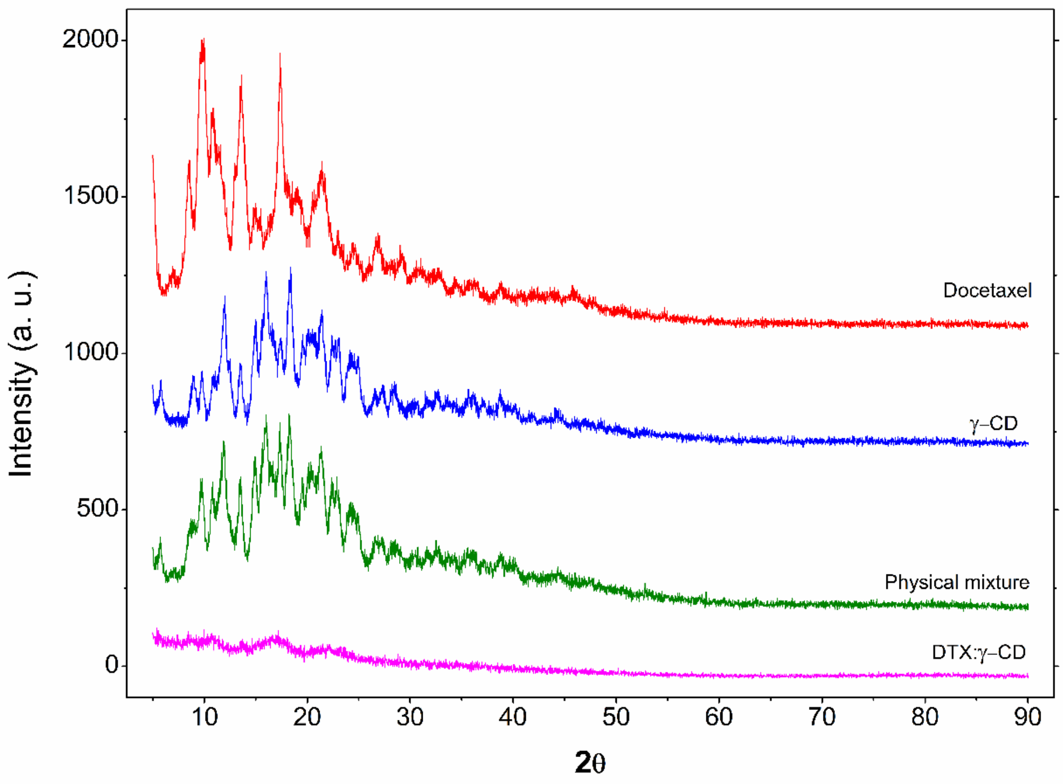

Figure S6. XRD diffractograms of DTX, γ-CD, physical mixture (1:2 mol%), and DTX:γ-CD inclusion complex (1:2 mol%).

Table S1 - Melting point and enthalpy values of excipients, physical mixture and DTX:γ-CD complex (data from Figure S5).

Sample	Melting Point (°C)	Enthalpy ($J.g^{-1}$)
DTX	166.1	9.55
γ-CD	203.7	129.70
Physical Mixture (1:2 mol%)	166.3,<br>200.2	6.60,<br>86.22
DTX:γ-CD complex (1:2 mol%)	136.6	23.02
